# Generation and application of river network analogues for use in ecology and evolution

**DOI:** 10.1002/ece3.6479

**Published:** 2020-06-30

**Authors:** Luca Carraro, Enrico Bertuzzo, Emanuel A. Fronhofer, Reinhard Furrer, Isabelle Gounand, Andrea Rinaldo, Florian Altermatt

**Affiliations:** ^1^ Department of Aquatic Ecology Swiss Federal Institute of Aquatic Science and Technology (Eawag) Dübendorf Switzerland; ^2^ Department of Evolutionary Biology and Environmental Studies University of Zurich Zürich Switzerland; ^3^ Department of Environmental Sciences, Informatics and Statistics University of Venice Ca' Foscari Venice Italy; ^4^ ISEM CNRS EPHE Université de Montpellier Montpellier France; ^5^ Department of Mathematics and Department of Computational Science University of Zurich Zürich Switzerland; ^6^ CNRS UPEC CNRS IRD INRA, Institut d’écologie et des sciences de l'environnement, IEES Sorbonne Université Paris France; ^7^ Laboratory of Ecohydrology Swiss Federal Institute of Technology in Lausanne (EPFL) Lausanne Switzerland; ^8^ Department of Civil, Environmental and Architectural Engineering University of Padua Padova Italy

**Keywords:** biodiversity, dispersal, ecological modeling, landscape, metacommunity, optimal channel network, river networks, spanning trees

## Abstract

1. Several key processes in freshwater ecology are governed by the connectivity inherent to dendritic river networks. These have extensively been analyzed from a geomorphological and hydrological viewpoint, yet structures classically used in ecological modeling have been poorly representative of the structure of real river basins, often failing to capture well‐known scaling features of natural rivers. Pioneering work identified optimal channel networks (OCNs) as spanning trees reproducing all scaling features characteristic of natural stream networks worldwide. While OCNs have been used to create landscapes for studies on metapopulations, biodiversity, and epidemiology, their generation has not been generally accessible.
2. Given the increasing interest in dendritic riverine networks by ecologists and evolutionary biologists, we here present a method to generate OCNs and, to facilitate its application, we provide the R‐package *OCNet*. Owing to the stochastic process generating OCNs, multiple network replicas spanning the same surface can be built; this allows performing computational experiments whose results are irrespective of the particular shape of a single river network. The OCN construct also enables the generation of elevational gradients derived from the optimal network configuration, which can constitute three‐dimensional landscapes for spatial studies in both terrestrial and freshwater realms. Moreover, the package provides functions that aggregate OCNs into an arbitrary number of nodes, calculate several descriptors of river networks, and draw relevant network features.
3. We describe the main functionalities of the package and its integration with other R‐packages commonly used in spatial ecology. Moreover, we exemplify the generation of OCNs and discuss an application to a metapopulation model for an invasive riverine species.
4. In conclusion, *OCNet* provides a powerful tool to generate realistic river network analogues for various applications. It thereby allows the design of spatially realistic studies in increasingly impacted ecosystems and enhances our knowledge on spatial processes in freshwater ecology in general.

## Graphical abstract

Recently, research on spatial dynamics in ecology and evolution has bloomed. However, spatial structures used in theoretical and empirical ecological studies are often not representative of realistic landscapes. This is particularly true for river networks, which are of paramount interest to ecologists owing to their wide (but currently severely declining) biodiversity. Indeed, most of ecological work has been neglecting the scaling character of real river networks, despite well‐established knowledge in the fields of geomorphology and hydrology. Here, we present a method to create optimal channel networks (OCNs, i.e., river network analogues reproducing all topographic and scaling features of natural rivers), and the respective R‐package allowing their generation and analysis. We review the theoretical background underlying the OCN concept, present the main package functionalities, discuss possible applications in the realm of ecology and evolution, and detail how the package can be integrated with other popular R‐packages in spatial ecology.

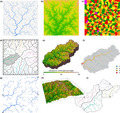

## 1 INTRODUCTION

The central goal of ecology is to causally understand patterns and processes in ecological systems, such as species coexistence, biodiversity patterns, or the unfolding of species invasions (Elton, 1958; Gause, 1934). Much of early ecological theory and empirical work has either focused on local patterns and dynamics or has taken a spatially implicit perspective. However, virtually all natural ecosystems are spatially structured, and the relevance of the spatial dimension on ecological systems can hardly be overestimated (Hanski & Gaggiotti, 2004; Holyoak, Leibold, & Holt, 2005; Levin, 1992). Consequently, over the last decades, ecologists have started to account for spatial processes on population and community dynamics as well as biodiversity. Theoretical, comparative, and experimental studies have increasingly been done in a spatially explicit perspective (e.g., Altermatt, Schreiber, & Holyoak, 2011; Cadotte & Fukami, 2005; Dale & Fortin, 2014; Gilarranz & Bascompte, 2012; Hanski & Ovaskainen, 2000; Holyoak et al., 2005), especially promoted by theories on metapopulation and metacommunity dynamics.

A direct consequence of this spatial approach to ecology is the need to describe and understand the spatial structure and layout of natural ecosystems. While initial models of spatial dynamics assumed spatially implicit networks of populations or communities (Levins, 1970), all natural ecosystems follow spatially explicit structures. These structures, such as those typically found in coral reefs and atolls, mountainous landscapes and their elevational gradients, or tidal pools, are shaped by general geophysical processes resulting in characteristic landscape structures. Arguably the most iconic (but also among the most widespread) landscape structure is found in riverine networks (Leopold, Wolman, & Miller, 1964; Rodriguez‐Iturbe & Rinaldo, 2001): erosional forces balancing uplift create dendritic networks of rivers and streams following universal patterns. These networks are characterized by their fractal, scale‐free structure, as well as by universally applicable laws regarding many geomorphological and hydrological variables of direct relevance to ecology, such as catchment area, river bed width and depth, or variation in discharge (Horton, 1945; Leopold & Maddock, 1953; Rodriguez‐Iturbe & Rinaldo, 2001). In contrast to these specific features of natural landscape structures, much of ecological and evolutionary theory and experiments, but also much of the species‐distribution modeling has assumed either random networks or simply structured linear, circular or Cartesian networks, in which local patches are connected to their 2, 4, or 8 nearest neighbors (e.g., Bascompte & Solé, 1996; Bell & Gonzalez, 2011; Holland & Hastings, 2008). This oversimplification of spatial network structures may limit the plausibility and relevance of the findings. An application to more realistic network structures has, however, often been hindered by the lack of formalized, spatially correct, and generalizable network structures as well as easily accessible tools generating them.

Riverine ecosystems are not only of high interest to ecologists due to their universal network structure, but also due to the considerable biodiversity inhabiting them (Altermatt, 2013; Altermatt et al., 2020; Balian, Segers, Lévèque, & Martens, 2008). River networks cover <1% of the landmasses, but contain up to 10% of all species. However, this high biodiversity, as well as the associated ecosystem functions, is threatened by various anthropogenically induced causes, including pollution, biological invasions, or damming and modification of the network structure (Darwall et al., 2018; Vörösmarty et al., 2010). An understanding of many of these processes requires a spatially explicit approach, such as how pollution and chemicals are transported in riverine networks (Helton, Hall, & Bertuzzo, 2018), how organisms spread along rivers and invade riverine ecosystems (Giometto, Altermatt, & Rinaldo, 2017; Mari, Casagrandi, Bertuzzo, Rinaldo, & Gatto, 2014), or how the modification of network structures across drainage basins affects local diversity (Leuven et al., 2009). Consequently, there has been a rapid increase in ecological and evolutionary studies considering the effect of river‐like network structures on ecological dynamics over the last two decades (Altermatt, 2013; Campbell Grant, Lowe, & Fagan, 2007; Fagan, 2002), paralleled by an increase in methodological tools to analyze such spatial datasets (Duarte et al., 2019; Muneepeerakul et al., 2008; Paz‐Vinas & Blanchet, 2015; Peterson et al., 2013; Rinaldo, Gatto, & Rodriguez‐Iturbe, 2020; Rodriguez‐Iturbe, Muneepeerakul, Bertuzzo, Levin, & Rinaldo, 2009; Welty, Torgersen, Brenkman, Duda, & Armstrong, 2015).

**FIGURE 1:**
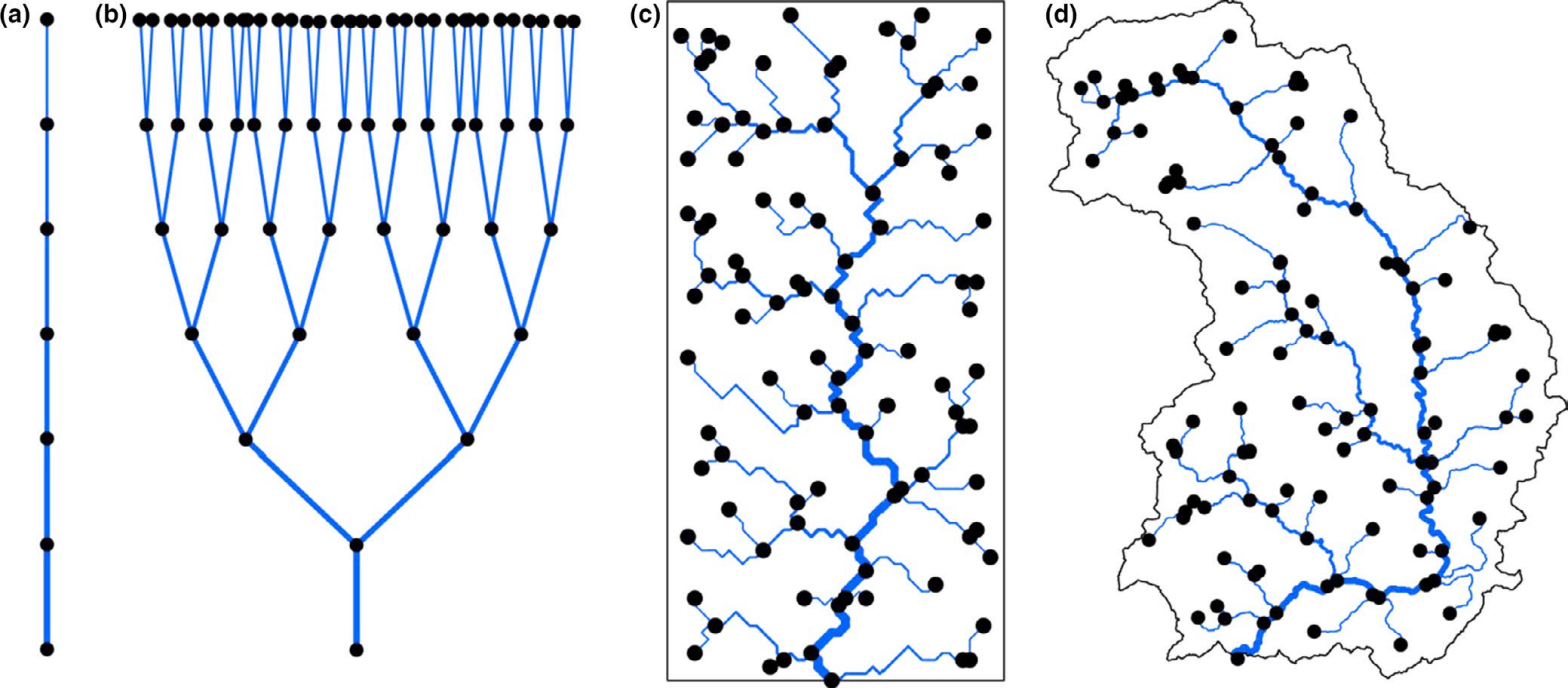
Examples of river network analogues with increasing level of resemblance with real river networks. Line width increases toward the downstream direction. (a) Linear array of patches (Vannote et al., 1980). (b) Binary‐fission‐like tree (Fagan, 2002; Paz‐Vinas et al., 2015). (c) An OCN spanning a 50 × 100 lattice, aggregated with a threshold area equal to 25 pixels. (d) A real river (Thur, Switzerland), spanning an area of 740 km^2^, extracted from a digital elevation model with a threshold area equal to 4 km^2^

While all of these works acknowledge the importance of studying rivers in a spatially explicit perspective, a large part of them is built on networks that do not factually capture many of the inherent characteristics of true riverine networks. Notable examples range from the River Continuum Concept (Vannote, Minshall, Cummins, Sedell, & Cushing, 1980), which describes rivers as a single, linear array of patches, to slightly more complex bifurcation networks or alterations thereof (Anderson & Hayes, 2018; Brown & Swan, 2010; Chaput‐Bardy, Fleurant, Lemaire, & Secondi, 2009; Fagan, 2002; Morrissey & De Kerckhove, 2009; Paz‐Vinas, Loot, Stevens, & Blanchet, 2015; Seymour & Altermatt, 2014; Seymour, Fronhofer, & Altermatt, 2015; Yeakel, Moore, Guimarães, & de Aguiar, 2014). All of these studies use networks that may at first sight look like “river” networks, but do not satisfy the necessary constraint posed by draining a given surface (Figure 1). Furthermore, these constructs do not adequately represent the connectivity and several geometric properties (like the distributions of upstream and downstream lengths, and of total contributing area at a point) inherent to natural river networks, and lack the space‐filling attribute of small to smallest streams not only incrementally flowing into larger streams, but also the common direct inflow of very small streams into large streams. As such, all of this work has been ignoring the extensive and long‐lasting knowledge from geomorphology that has appropriately acknowledged and formalized the spatial unfolding of dendritic river networks.

In particular, the fractal character of river networks, epitomized by Horton's laws (Horton, 1945) on bifurcation and length ratios, was observed with regard to several morphological and hydrological characteristics of river basins and expressed by means of a number of power‐law relationships, which are the signatures of fractal behavior (Mandelbrot, 1983; Maritan, Rinaldo, Rigon, Giacometti, & Rodríguez‐Iturbe, 1996). Notable examples are Hack's law (Hack, 1957) $L\sim A^{h}$, linking the maximum upstream channelized length *L* at any location in the river with the corresponding drainage area *A*; the slope–area relationship $s\sim A^{\gamma -1}$, where *s* is the channel slope (Tarboton, Bras, & Rodriguez‐Iturbe, 1989); the scaling of the probability distribution of drainage areas $P\left(A\geq a\right)\sim a^{-\beta }$ (Rodriguez‐Iturbe, Ijjász‐Vásquez, Bras, & Tarboton, 1992). Typical values observed in real rivers for the scaling exponents are h≈0.57, γ≈0.5, and β≈0.43 (Rinaldo, Rigon, Banavar, Maritan, & Rodriguez‐Iturbe, 2014). From a hydraulic geometry viewpoint, Leopold's relationships (Leopold & Maddock, 1953) express how mean river depth, width, and velocity change, both at‐a‐station and along the river's course, as power‐law functions of discharge, which in turn scales linearly with drainage area (strictly speaking, this applies to landscape‐forming discharges; Rodriguez‐Iturbe & Rinaldo, 2001).

Such scale‐invariant properties of river networks prompted the development of a model of idealized stream networks: optimal channel networks (OCNs). OCNs are “optimal” inasmuch as their configuration corresponds to a minimum of total energy expenditure and reproduces all scaling features of real rivers (Maritan et al., 1996; Rinaldo et al., 1992, 2014; Rodriguez‐Iturbe, Rinaldo, et al., 1992). Importantly, OCNs are exact stationary solutions of the general equation describing landscape evolution (Banavar, Colaiori, Flammini, Maritan, & Rinaldo, 2001). The OCN construct allows the generation of an unlimited number of different network replicas spanning the same drainage domain, therefore enabling one to run computational experiments and derive results that are independent of the shape of a single river network, which would not be the case if real rivers were used as landscapes. Moreover, OCNs enable the investigation of spatial processes occurring not only in dendritic river networks, but also along the elevational gradients of fluvial landscapes (Bertuzzo et al., 2016; Giezendanner, Bertuzzo, Pasetto, Guisan, & Rinaldo, 2019). To this regard, it is worthwhile to note that the elevational landscape generated by an OCN is such that the graph obtained by following the steepest descent directions reproduces the OCN structure (Balister et al., 2018).

OCNs have been used to investigate a number of ecological issues, ranging from metapopulation structure in riverine (Bertuzzo, Rodriguez‐Iturbe, & Rinaldo, 2015; Fronhofer & Altermatt, 2017; Mari et al., 2014) and terrestrial landscapes (Bertuzzo et al., 2016; Giezendanner et al., 2019); habitat fragmentation (Sarker, Veremyev, Boginski, & Singh, 2019); spreading of human (Bertuzzo, Casagrandi, Gatto, Rodriguez‐Iturbe, & Rinaldo, 2010; Gatto et al., 2013; Mari, Casagrandi, Bertuzzo, Rinaldo, & Gatto, 2019) and animal (Carraro, Mari, Gatto, Rinaldo, & Bertuzzo, 2018) waterborne pathogens; ecosystem processes, such as carbon (Bertuzzo, Helton, Hall, & Battin, 2017; Koenig et al., 2019) and nitrogen cycling (Helton et al., 2018); migration fronts of human populations (Campos, Fort, & Méndez, 2006); cross‐ecosystem subsidies (Harvey, Gounand, Fronhofer, & Altermatt, 2020); sampling strategies for environmental DNA in rivers (Carraro, Stauffer, & Altermatt, 2020); riverine biodiversity patterns from a theoretical viewpoint (Muneepeerakul, Bertuzzo, Rinaldo, & Rodriguez‐Iturbe, 2019); or by means of mesocosm experiments (Carrara, Altermatt, Rodriguez‐Iturbe, & Rinaldo, 2012; Carrara, Rinaldo, Giometto, & Altermatt, 2014; Harvey, Gounand, Fronhofer, & Altermatt, 2018).

Despite the long‐standing establishment of the OCN concept, its application especially in ecology and evolutionary biology has been lagging behind, likely because easily accessible code or appropriate tools have been lacking. This is particularly regrettable considering the recent bloom of tools allowing the statistical analysis of data from real dendritic networks (e.g., the R‐package SSN; Ver Hoef, Peterson, Cliord, & Shah, 2014). However, such tools are specifically designed for real river networks, while their applicability to virtually generated networks is limited. To fill this gap, we here describe the methodological and mathematical frameworks that underly *OCNet*, an R‐package for the generation and analysis of optimal channel networks, and provide guidelines and examples to facilitate the use of this tool.

## 2 THE OCNET PACKAGE

The OCN concept is based on the assumption that river network configurations occurring in nature correspond to a minimum of total energy dissipation across the landscape. Both this assumption and the ensuing algorithm generating OCNs are well supported by a comparison with river networks globally (Rinaldo et al., 1992, 2014; Rodriguez‐Iturbe, Rinaldo, et al., 1992). This section is structured as follows: first, we provide an overview on the theoretical background underlying the generation of an OCN; second, we outline the structure of the *OCNet* package; third, we clarify some concepts concerning the various aggregation levels at which an OCN can be defined and used.

### 2.1 Theoretical background

Let us consider a regular lattice made up of *N* cells, where each cell represents the generic node *i* of the network. Each node *i* is connected via a link to one of its nearest neighbors. The energy dissipation across the *i*th network link is proportional to $Q_{i}\Delta h_{i}$, where *Q_i_* is the landscape‐forming discharge in the link (Rinaldo et al., 2014), and $\Delta h_{i}=s_{i}L_{i}$ the corresponding elevation drop, with *s_i_* identifying slope and *L_i_* link length. By assuming $Q_{i}\sim A_{i}$ (Rodriguez‐Iturbe & Rinaldo, 2001), where *A_i_* is the area drained by link *i*, and the slope–area relationship $s_{i}\sim A_{i}^{\gamma -1}$ (Tarboton et al., 1989), the functional representing total energy expenditure across a landscape formed by *N* cells reads.

(1) $$H=\sum_{i=1}^{N}A_{i}^{\gamma }.$$

Note that link lengths do not appear in the above formula, as they can be considered constant with no loss of generality. The OCN configuration is defined by an adjacency matrix **W** whose corresponding set of drainage areas $A=\left[A_{1},\ldots ,A_{N}\right]$ yields a local, dynamically accessible minimum of Equation (1). Note that the correspondence between **A** and the adjacency matrix **W** of a tree is subsumed by the relationship $\left(I_{N}-W^{T}\right)A=1$, where **I** *_N_* is the identity matrix of order *N*, and **1** a *N*‐dimensional vector of ones (Bertuzzo et al., 2015).

**FIGURE 2:**
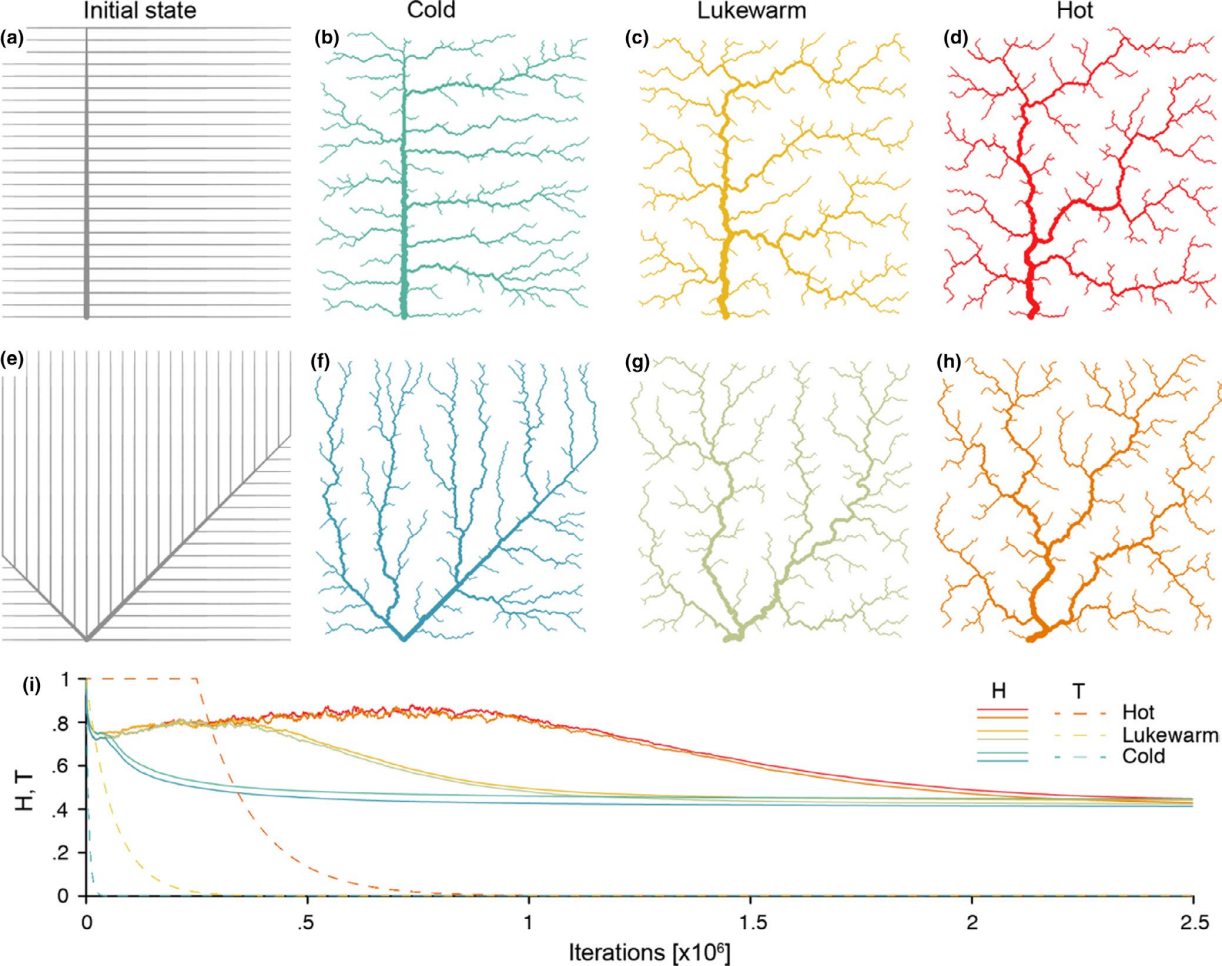
(a)Effect of initial network state (rows) and cooling schedule (columns) on the final OCN configuration. (b, c, d) OCNs on 250 × 250 lattices generated from the initial state shown in panel (a. f, g, h) As above but with initial state as shown in panel e. (i) Dynamics of total energy expenditure H (Equation (1)) and temperature T (i.e., cooling schedule of the simulated annealing algorithm) for the 6 OCNs displayed above. Values of H and T are normalized by the energy of the initial network state. Note that, for graphical reasons, the initial states shown in panels (a, e) refer to 25 × 25 lattices

Minimization of Equation (1) is operated by means of a simulated annealing technique: starting from a feasible initial flow configuration (i.e., a spanning tree, see Figure 2a,e), a link at a time is rewired to one of its nearest neighbors; if the obtained configuration is a spanning tree, *H* is computed; the new configuration is accepted if it lowers total energy expenditure; if this is not the case, the new configuration can still be accepted with a probability controlled by the cooling schedule of the simulated annealing algorithm. Such myopic search, which only explores close configurations, actually mimics the type of optimization that nature performs, at least in fluvial landscapes (Rinaldo et al., 2014). Notably, restricting the search of a network yielding a minimum of Equation (1) to spanning, loopless configurations entails no approximation, because every spanning tree is a local minimum of total energy dissipation (Banavar, Colaiori, Flammini, Maritan, & Rinaldo, 2000). The shape of the so‐obtained OCN retains the heritage of the initial flow configuration, although the extent to which this occurs is partly controlled by the cooling schedule adopted (Figure 2). This underpins the concept of feasible optimality, that is, the search for dynamically accessible configurations.

### 2.2 Overall setup of the package

The *OCNet* package consists of a series of functions that allow constructing river‐analogue networks as well as calculating a number of metrics and descriptors commonly used in spatial ecology. The networks constructed by the package are built at several levels of aggregation. At each level, they are generally defined by a number of nodes, an adjacency matrix, a vector of contributing areas and two vectors with longitudinal and latitudinal coordinates of the nodes. The functions constituting the *OCNet* package are intended to be applied in sequential order, and the respective output can be directly used to visualize the created networks and linked to other commonly used R‐packages.

**FIGURE 3:**
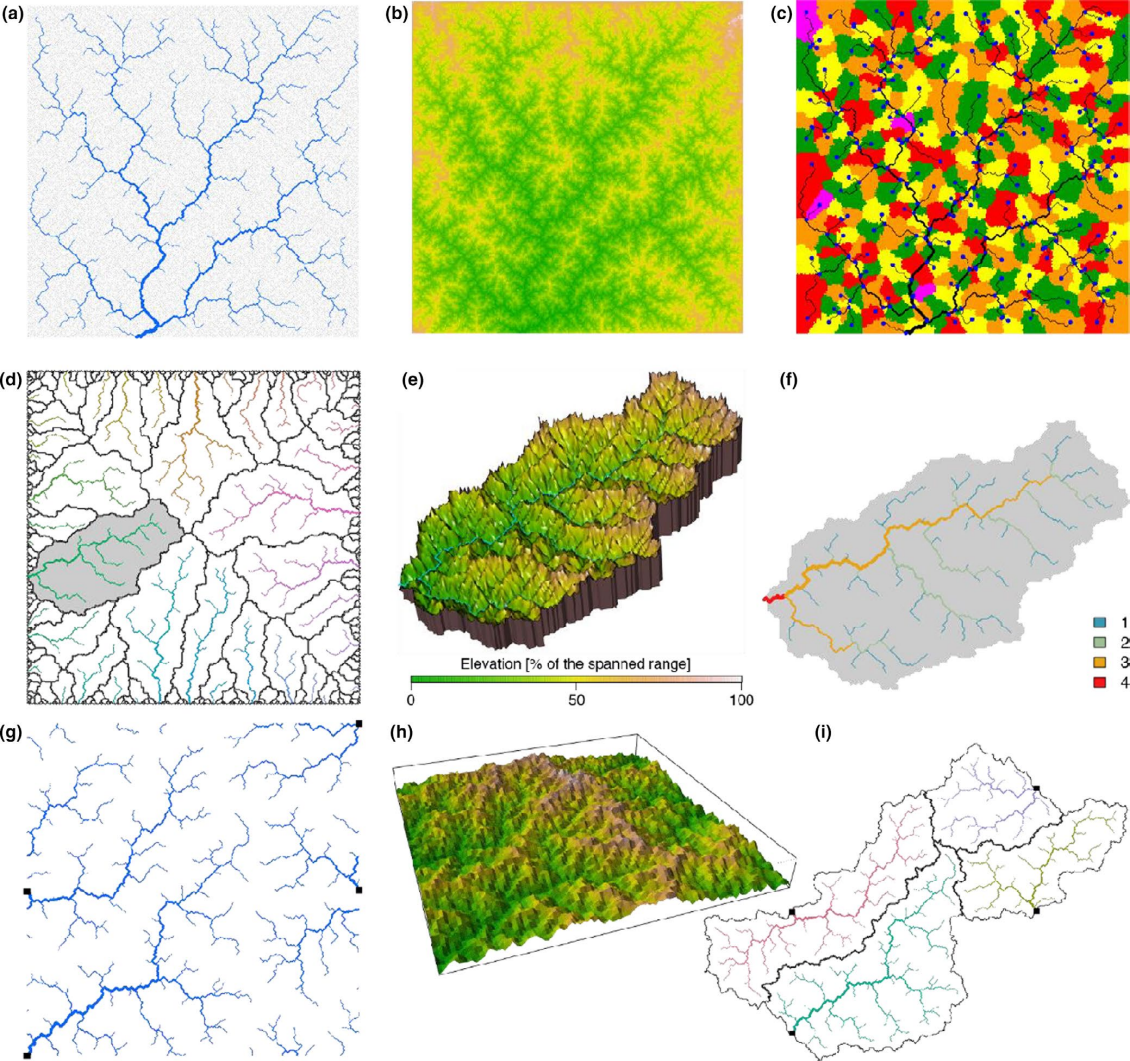
Examples of outputs from *OCNet*'s graphical functions. (a) Representation of an OCN generated on a 250 × 250 lattice (*draw_simple_OCN*). Note that the network spans the whole lattice; for graphical reasons, the portion of network exceeding a given *A_T_* is plotted in blue. (b) Planar representation of the elevational landscape generated by the OCN of panel a (*draw_elev2D_OCN*). (c) Partitioning of the lattice into subcatchments for the OCN of panel a (*draw_subcatchments_OCN*); blue dots indicate locations of the nodes at the AG level. (d) Representation of an OCN generated on a 400 × 400 lattice, with all perimetral pixels as outlets (*draw_contour_OCN*); black solid lines display partitioning among catchments; the gray background identifies the largest catchment. (e) 3D representation of the largest catchment within the OCN of panel (d) (*draw_elev3Drgl_OCN*). (f) Strahler stream order values for the largest catchment within the OCN of panel (d) (*draw_thematic_OCN*). (g) Representation of an OCN generated on a 300 × 300 lattice, with 4 outlets (shown by black squares) and periodic boundaries (*draw_contour_OCN*). (h) Perspective 3D representation of the OCN of panel (g) (*draw_elev3D_OCN*). (i) Real‐shaped representation of the OCN of panel (g) (*draw_contour_OCN*)

The first function, *create_OCN*, only requires the longitudinal and latitudinal dimensions of the lattice as necessary inputs, while several other parameters can be optionally tuned to obtain customized results. Some examples are provided in the following section; extensive further information is given in the package documentation. The output of *create_OCN* is a list containing a sublist termed FD that, in turn, encloses key information on the topology of the network, among which the adjacency matrix (written in sparse form via the *spam* format (Furrer & Sain, 2010)) and a vector of contributing areas. The subsequent functions *landscape_OCN* (generation of the three‐dimensional landscape derived from the network configuration), *aggregate_OCN* (aggregation of the OCN at various levels—see *Aggregation levels*), *paths_OCN* (evaluation of paths among network nodes, and lengths thereof), *rivergeometry_OCN* (hydraulic geometry of the river network, following Leopold and Maddock (1953)) require as necessary input the output list produced by the previous function, in the aforementioned order (except *rivergeometry_OCN*, which can be executed after *aggregate_OCN*). The output of these functions is a list where all objects of the input list are copied, and to which new objects are added. Note that output lists contain all input values, to avoid inconsistencies in the sequential application of functions. A group of functions (identified by the prefix “*draw_*”, see examples in Figure 3) are devoted to graphical representations of the OCN.

### 2.3 Aggregation levels

**FIGURE 4:**
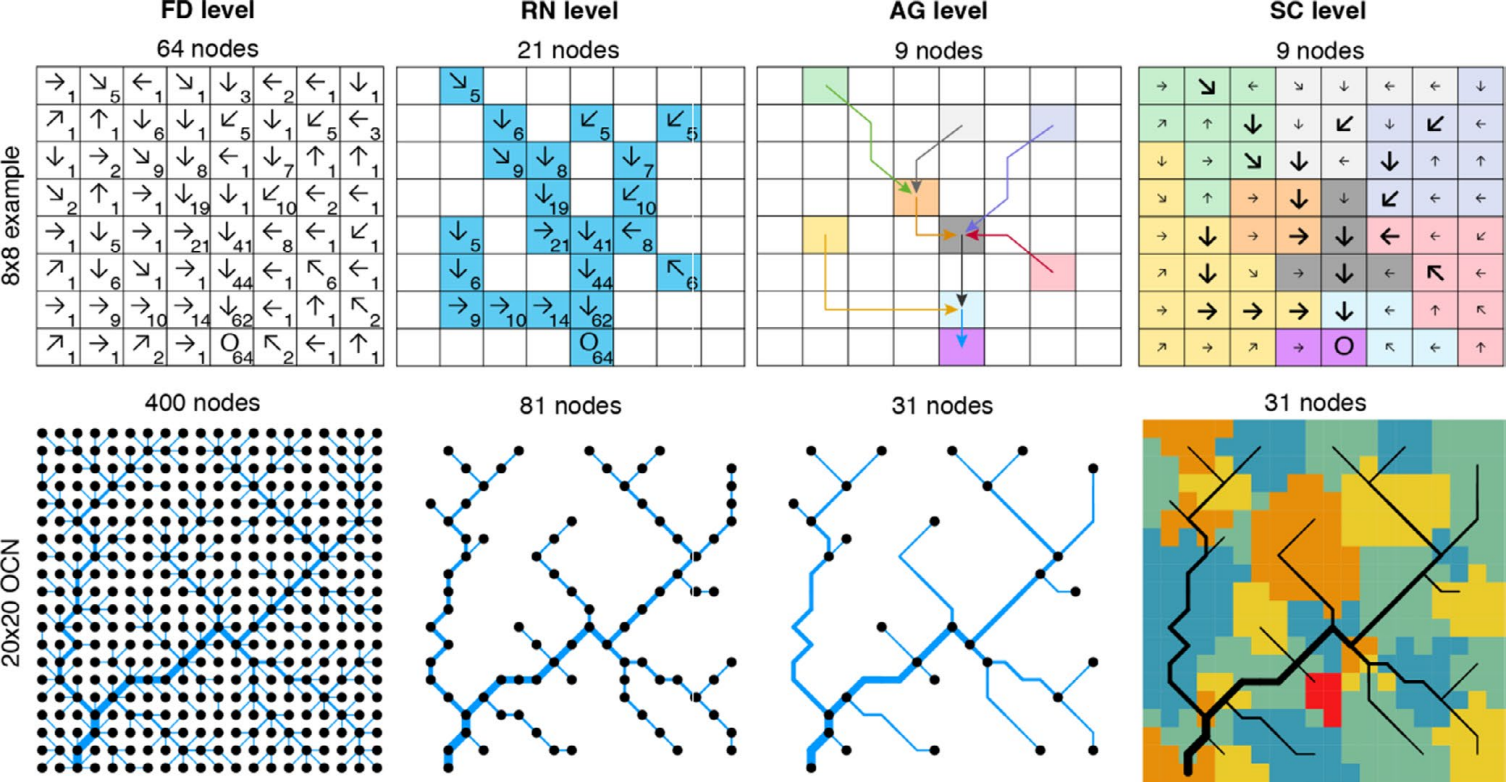
Representation of the different aggregation levels. Top row: example from a single‐outlet 8 × 8 lattice describing how the aggregation procedure operated by *aggregate_OCN* works. Letter ‘O’ identifies the outlet pixel. Arrows on the other pixels identify flow directions; note that the these are not representative of an OCN, but are here presented only for illustrative purposes. Numbers represent the cumulative drainage area (in number of pixels). At the FD level, all 64 pixels belong to the network. To obtain the RN level, a threshold area *A_T_* = 5 pixels is applied to distinguish pixels belonging to the river network. Bottom row: the same procedure is applied to a single‐outlet, 20 × 20 OCN (obtained via the script presented in *Generation of an OCN*). Aggregation is performed with *A_T_* = 5 pixels. Note that river width is proportional to the square root of drainage area (Leopold & Maddock, 1953)

Before moving to the illustration of some possible applications of the package, we here clarify some concepts and terminology with respect to the aggregation of OCNs. Additional details are provided in the package documentation. Networks produced by the OCN algorithm can be used in a variety of fashions (see Table 1 for a review) by exploiting different connectivity metrics that are embedded in the OCN construct. At a first, nonaggregated level, each cell of the lattice (also termed as pixel) constitutes a node of the network (see Figure 4), and the connectivity among nodes is ruled by the flow direction pattern (represented by the adjacency matrix) obeying the OCN principle. This is here referred to as the flow direction level (FD).

**TABLE 1:** Types of OCN aggregation schemes used in previous studies

Aggregation	References
N4/N8	Bertuzzo et al. (2016) (N4 neighbourhood); Giezendanner et al. (2019) (dispersal kernel)
FD	Bertuzzo et al. (2010); Bertuzzo et al. (2015); Campos et al. (2006); Gatto et al. (2013); Mari et al. (2014); Muneepeerakul et al. (2019); Sarker et al. (2019)
RN	Bertuzzo et al. (2017); Carraro et al. (2020)
AG	Carrara et al. (2012), Carrara et al. (2014); Carraro et al. (2018); Carraro et al. (2020); Fronhofer and Altermatt (2017); Harvey et al. (2018); Harvey et al. (2020); Helton et al. (2018); Koenig et al. (2019); Mari et al. (2019)
SC	Helton et al. (2018)

As customary in hydrology when extracting a river network based on digital elevation models of the terrain (O'Callaghan & Mark, 1984), a threshold *A_T_* on drainage area can be imposed to identify those pixels of the lattice that constitute nodes of the river network (RN, second level—see Terui et al. (2018) for an example of an ecological application of non‐OCN synthetic networks akin to OCNs at the RN level). In a third, aggregated level (AG), nodes correspond to sources, confluences and outlet(s) of the river network identified at the RN level. The whole lattice is then partitioned into areas that directly drain into the nodes at the AG level, or the edges departing from them, thereby constituting the fourth, subcatchment level (SC).

A fifth level (catchment, CM) partitions the lattice into regions that are drained by different outlets, when the multiple‐outlet option in *create_OCN* is enabled (see following section). Finally, in an optional, zero‐level spatial structure, all lattice pixels are treated as nodes, but connectivity follows the Von Neumann (4 nearest neighbors, level N4) or Moore (8 nearest neighbors, level N8) neighborhoods, as in the green network described by Altermatt (2013). In this case, the OCN was used to generate a realistic elevation gradient governed by fluvial erosion, on which, for instance, the structure of terrestrial metapopulations can be studied (Bertuzzo et al., 2016; Giezendanner et al., 2019).

The final output of the *OCNet* functions is a list of lists, each of which named after the corresponding aggregation level (N4/N8, FD, RN, AG, SC, CM) and containing relevant topological and morphological information for that level. Variables may vary in number, type, and definition among the sublists, although the adjacency matrix is defined for all levels, while the drainage area vector is defined for all levels but N4/N8.

## 3 OVERVIEW OF PACKAGE FEATURES

### 3.1 Number of outlets, boundary types, and elevational gradients

Although the OCN principle is primarily intended to be applied to networks spanning the whole drainage domain (where the area drained by the outlet is equal to the lattice area, see an example in Figure 3a–c), the generalization to the case of multiple networks—each of which subsumed by a different outlet—within the same lattice is straightforward. Indeed, the very same mathematical formulation presented in *Theoretical background* holds when multiple‐outlet pixels are imposed. Technically, this is done by preventing these pixels to drain into their neighboring pixels. In this case, the sum of the areas drained by all outlet pixels is equal to the lattice area. In the package, the multiple‐outlet option can be activated by means of the optional input *nOutlet* of function *create_OCN*. In the limiting case, all pixels at the lattice boundary can be treated as outlets (Bertuzzo et al., 2017; Sun, Meakin, & Jøssang, 1994). This is done by setting *nOutlet = “All”* in *create_OCN*. A graphical representation of an OCN obtained for the latter case is shown in Figure 3d.

When a pixel's flow direction is rewired during the search for an optimal network configuration, possible directions are generally those toward the eight neighboring pixels. This is not the case for the outlet pixels (which cannot be rewired) and the pixels at the lattice boundaries, which can be rewired to either three (corner pixels) or five (side pixels) neighboring cells. This latter assumption can be relaxed by allowing pixels at the boundary to drain into eight neighbors, by also considering pixels at the opposite sides as feasible directions. In *OCNet*, periodic boundaries can be enabled via the optional input *periodicBoundaries* of *create_OCN*. An example is shown in Figure 3g–i. Such option can be useful when the OCN lattice is to be considered as the periodic unit of an infinite landscape (Bertuzzo et al., 2016; Giezendanner et al., 2019), or when one aims at building OCNs spanning domains that are not lattice‐shaped (see Figure 3f,i).

Once an OCN has been created by the simulated annealing algorithm, the iterative application of the slope–area relationship starting from the outlet node and moving in the upstream directions enables the derivation of the elevation field subsumed by the OCN (up to two constants, e.g., the elevation and slope of the outlet pixel). Some examples of elevational landscapes built on OCNs are provided in Figure 3b,e,h. Importantly, the slope–area relationship only holds for the channeled portion of the domain, which implies, strictly speaking, that the OCN must not be aggregated if one aims at making use of a three‐dimensional landscape generated by an OCN. Moreover, the slope–area relationship is actually multiscaling (Tarboton et al., 1989), therefore the simple recursive application of $s_{i}\sim A_{i}^{\gamma -1}$ (as performed by the function *landscape_OCN*) to yield an elevational landscape is to be considered as a first approximation, suitable for ecological applications. Methods to account for the scaling of the variance of the slope–area relationship exist (Grimaldi, Teles, & Bras, 2005), but are beyond the scope of this work.

### 3.2 Relationship between threshold drainage area and number of nodes

Owing to the somewhat heuristic procedure for the definition of an aggregated network based on a threshold drainage area value *A_T_*, it is not possible to establish a priori how many nodes at the AG level correspond to a given *A_T_*. This in fact depends on the configuration of the OCN at the FD level, which is the result of a stochastic process. This issue is particularly relevant when OCNs are used in experiments where practical reasons enforce a limitation on the number of nodes that can be handled, or when several OCN replicas with the same number of aggregated nodes are required.

To help overcome this issue, *OCNet* includes the function *find_area_threshold_OCN*, which requires as input a nonaggregated OCN (produced by *landscape_OCN*) and evaluates the number of nodes resulting from the aggregation procedure for different values of *A_T_*. Such a function can therefore be used prior to *aggregate_OCN* to assess which threshold has to be used to obtain a network with the desired aggregation structure. Additionally, *find_area_threshold_OCN* also evaluates other variables that help characterize the network structure from a hydrological perspective, such as maximum stream order and drainage density. Maximum stream order can be of interest in some studies, when patch sizes need to be related to the structure of the underlying network but only few discrete dimensions are available, so that it is convenient to employ different patch sizes for different stream order values of the corresponding nodes (e.g., Harvey et al., 2018). Drainage density is relevant because it allows the assessment of hydrological characteristics of the aggregated OCN for a given metric resolution (i.e., the length in meters attributed to a pixel length—corresponding to the optional input *cellsize* of *create_OCN*), such as aridity and timing of the hydrologic response (Pallard, Castellarin, & Montanari, 2009).

**FIGURE 5:**
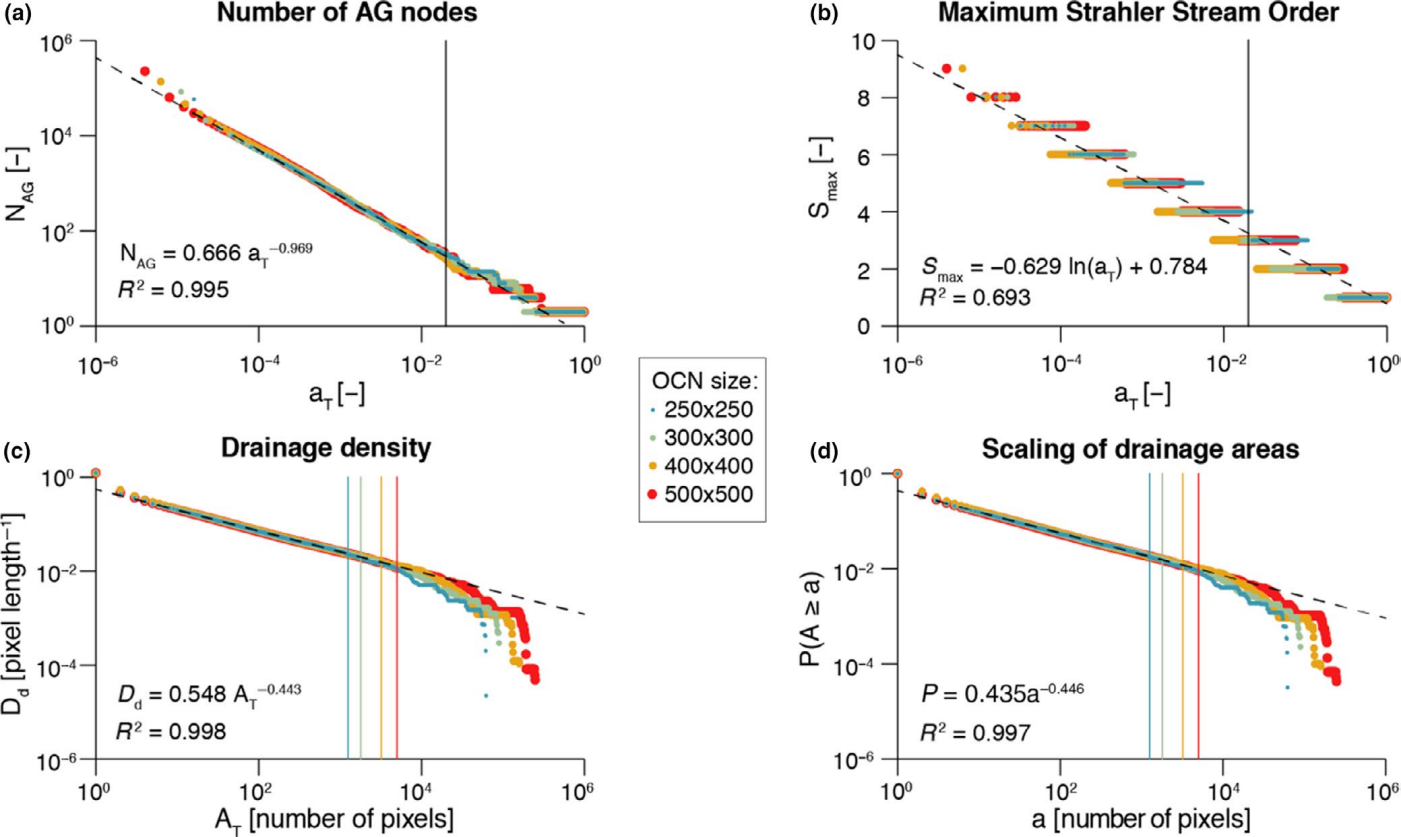
Effect of choice of threshold area *A_T_* on OCN configuration. Aggregation of four large OCNs is performed $\forall A_{T}=\left\{1,\ldots ,N\right\}$ via function *find_area_threshold_OCN*. For all panels, vertical lines indicate the cutoff value $A_{T}=0.02\cdot N$; only points corresponding to threshold area values below the cutoff are used to estimate the (dashed) regression lines, whose equations and *R* ^2^ values are reported. (a) Number of nodes at the AG level scales as a power‐law function of the normalized threshold area $a_{T}=A_{T}/N$. (b) Maximum Stahler stream order value as a function of normalized threshold area *a_T_*. (c) Drainage density scales as a power‐law function of threshold area *A_T_*. (d) Scaling behavior of OCNs: probability $P\left(A\geq a\right)$ of randomly sampling a pixel within the lattice whose drainage area *A* is not greater than a given value *a*

Figure 5 shows results from the application of *find_area_threshold_OCN* to several OCNs built on large lattices. When *A_T_* is lower than 2% of the lattice size, the number of nodes scales fairly well as a power law of the normalized threshold area (Figure 5a). This relationship allows qualitatively assessing the relevant range of *A_T_* corresponding to a sought number of nodes at the AG level, which can be used as input in *find_area_threshold_OCN* to speed up its execution, especially for large networks. Scaling relationships with *A_T_* are also found for maximum Strahler stream order (Figure 5b) and drainage density (Figure 5c). To provide an example, if a threshold *A_T_* = 20 pixels is applied to a 200 × 200 OCN, the expected number of nodes at the AG level is 1,052.4, the expected maximum stream order is 5.56, and the expected drainage density is *D_d_* = 0.1454 inverse planar units, which corresponds to a relatively wet catchment (*D_d_* = 2.91/km) of area 100 km^2^ (when 1 planar unit represents 50 m), or to a rather arid catchment (*D_d_* = 1.46/km) of area 400 km^2^ (if 1 planar unit is equal to 100 m). Notably, the relationship between drainage density and threshold area *A_T_* mirrors the scaling behavior of drainage areas (Figure 5d), which is characterized by an exponent *__* in the range [0.42;0.45] (see *Introduction* and Rinaldo et al. (2014)). Indeed, drainage density for a given *A_T_* is roughly (i.e., if differences in lengths between vertical/horizontal and diagonal flow directions are neglected) equal to the number of pixels whose area is greater than or equal to *A_T_*. Figure 5d also represents how the number of nodes at the RN level (*N*_RN_) scales with varying *A_T_*: to this end, it suffices to replace *a* with *A_T_* and $P\left(A\geq a\right)$ with $N_{\text{RN`}}/N$.

The scaling behavior of OCNs displayed in Figure 5 can also provide useful information with respect to the choice of values of relevant parameters *N* and *A_T_* that allow generating an OCN of adequate size for practical applications. To this extent, it is worthwhile to note that the OCN construct is invariant under coarse graining (Rinaldo et al., 2014; Rodriguez‐Iturbe & Rinaldo, 2001), which means that the choice of the lattice dimension *N* does not affect the scaling of drainage areas. As in the example above, such choice should rather be based on geomorphological arguments, that is, the answers to the questions: What is the area that the OCN is supposed to drain? What are the expected values of maximum stream order and drainage density on this area? However, as a rule‐of‐thumb indication, we suggest to perform aggregation with a threshold not greater than $A_{T}=0.02\cdot N$, such that the obtained configuration is not affected by the finite‐size scaling effect; this corresponds to an expected *N*_AG_  ≥  30 (see Figure 5a).

### 3.3 Compatibility with existing R‐packages

**FIGURE 6:**
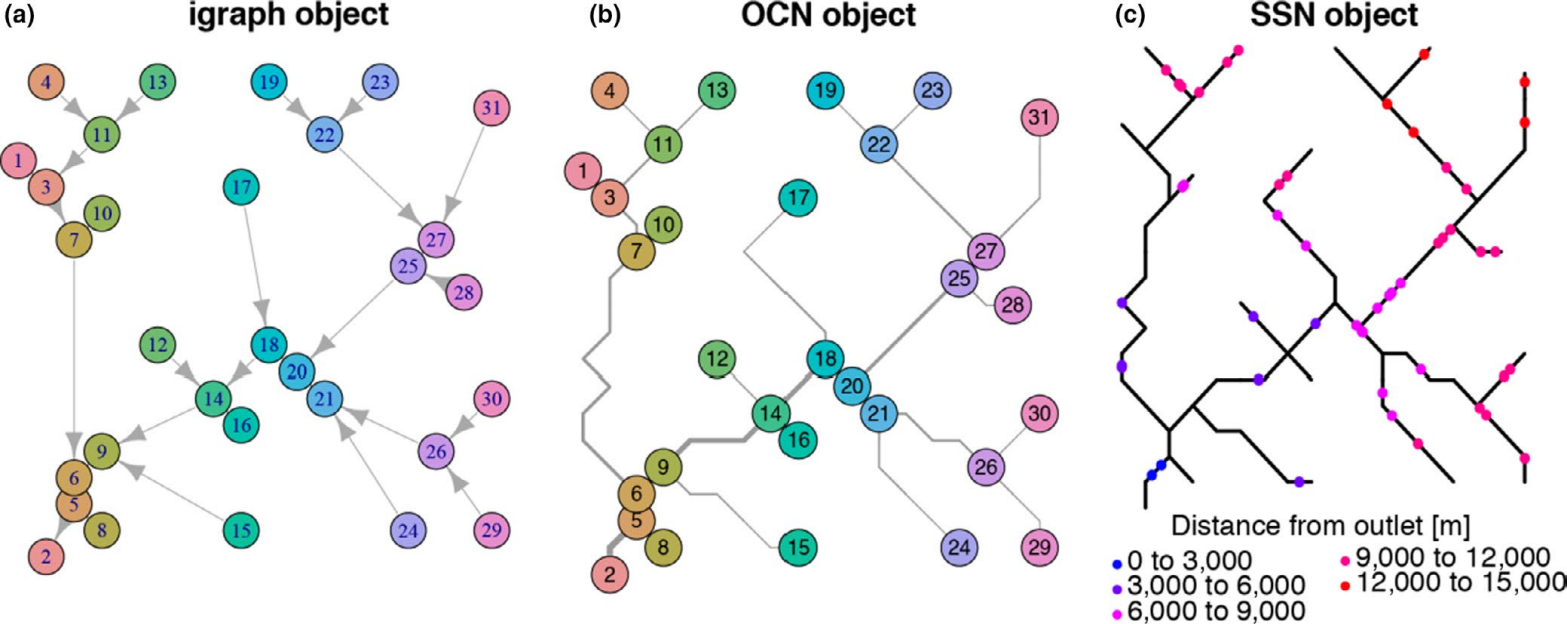
Compatibility of *OCNet* with packages *igraph* and *SSN*. Examples are built on the OCN obtained in *Generation of an OCN*. (a) The OCN, aggregated at the AG level, is transformed into an *igraph* object (via *OCN_to_igraph*), and plotted via the function *plot.igraph* of *igraph*. (b) The same OCN is plotted via *draw_thematic_OCN*. (c) The same OCN is transformed into an *SSN* object (via *OCN_to_SSN*) and plotted via the function *plot.SpatialStreamNetwork* of *SSN*. Here, 50 observation points have been sampled along the network by means of a binomial design, and their distance from the outlet is displayed

Specific functions of *OCNet* enable transformation of OCNs into objects that can be used by other commonly used R‐packages in spatial ecology. In particular, compatibility with *igraph* (Csardi & Nepusz, 2006), a package for network analysis and visualization, is provided by function *OCN_to_igraph*. Moreover, function *OCN_to_SSN* transforms an OCN at a desired aggregation level into an object that can be read by *SSN*, a package on spatial statistical modeling and prediction for data on stream networks (Ver Hoef et al., 2014). Examples for these functions are shown in Figure 6. Finally, output from *OCNet* can be used in combination with R‐packages for geostatistical modeling such as *gstat* (Pebesma, 2004), based on the coordinates of nodes of an OCN given at any aggregation level. Remarkably, adjacency matrices and other information can easily be extracted as base R objects, which guarantees compatibility with virtually every R‐package and even other programming languages.

## 4 APPLICATION EXAMPLE: A METAPOPULATION MODEL

In order to show a possible application of the *OCNet* package, we now apply a simple metapopulation model for an invasive riverine species to an OCN.

### 4.1 Generation of an OCN

Let us build an OCN with the following assumptions: it spans a 20 × 20 lattice, with a single outlet located close to the southwestern corner of the lattice, and each pixel represents a square of side 500 m (total size of the catchment is therefore 100 km^2^); the elevation, slope and channel width of the outlet node are 0 m a.s.l., 0.01, and 5 m, respectively; the threshold area used to aggregate the network is equal to 1.25 km^2^. The code lines to build such network are the following:

set.seed(1) # use fixed random number generator.

OCN <‐ create_OCN(20, 20, outletPos = 3, cellsize = 500).

OCN <‐ landscape_OCN(OCN, slope0 = 0.01).

OCN <‐ aggregate_OCN(OCN, thrA = 1.25e6).

OCN <‐ paths_OCN(OCN, pathsRN = TRUE).

OCN <‐ rivergeometry_OCN(OCN, widthMax = 5).

The resulting OCN is shown in Figure 4.

### 4.2 Metapopulation model

Let us build a discrete‐time, deterministic metapopulation model on the previously built OCN, according to the following assumptions: (a) the model is run on the OCN aggregated at the RN level (consisting of *N_n_* nodes); (b) population growth at each node follows the Beverton‐Holt model (Beverton & Holt, 1957), with baseline fecundity rate *r* = 1.05 constant for all nodes, and carrying capacity $K_{i}=10\cdot W_{i}$, where *W_i_* is the river width of the network node *i*; (c) at each time step *t*, the number of individuals moving from node *i* is equal to $gP_{i}\left(t\right)$, where *g* = 0.1 is a mobility rate constant for all nodes, and $P_{i}\left(t\right)$ is the (expected) population size at node *i* and time *t*; (d) at each time step, individuals at node *i* can only move to a node that is directly connected to *i*, either downstream or upstream; (e) *p_d_* and $p_{u}=1-p_{d}$ identify the probability to move downstream or upstream, respectively; (f) if the indegree of node *i* is larger than 1 (namely the node has multiple upstream connections), individuals moving upstream are split among the possible destination nodes into fractions *Y_i_* proportional to their drainage areas; (g) as initial condition, all network nodes are uninhabited barring the node *f* that is farthest from the outlet (where $P_{f,1}=1$). The model equation hence reads.

$$P_{i,t+1}=\frac{rP_{i,t}}{1+\left(r-1\right)P_{i,t}/K_{i}}+g\left[p_{d}\sum_{j=1}^{N_{n}}w_{\mathrm{ji}}P_{j,t}+p_{u}Y_{i}\sum_{j=1}^{N_{n}}w_{\mathrm{ij}}P_{j,t}-\left(p_{d}D_{i}+p_{u}U_{i}\right)P_{i,t}\right],$$

where *w_ij_* is a generic entry of the adjacency matrix **W** expressing OCN connectivity at the RN level; *D_i_* (*U_i_*) is equal to one if there is a downstream (upstream) connection available from node *i* and is null otherwise. Weights *Y_i_* are defined as

$$Y_{i}=\frac{A_{i}}{\sum_{k=1}^{N_{n}}w_{ki^{\prime }}A_{k}},$$

where *i*′ is such that *w_ii′ _ = 1*, and *A_i_* is the drainage area at node *i*.

**FIGURE 7:**
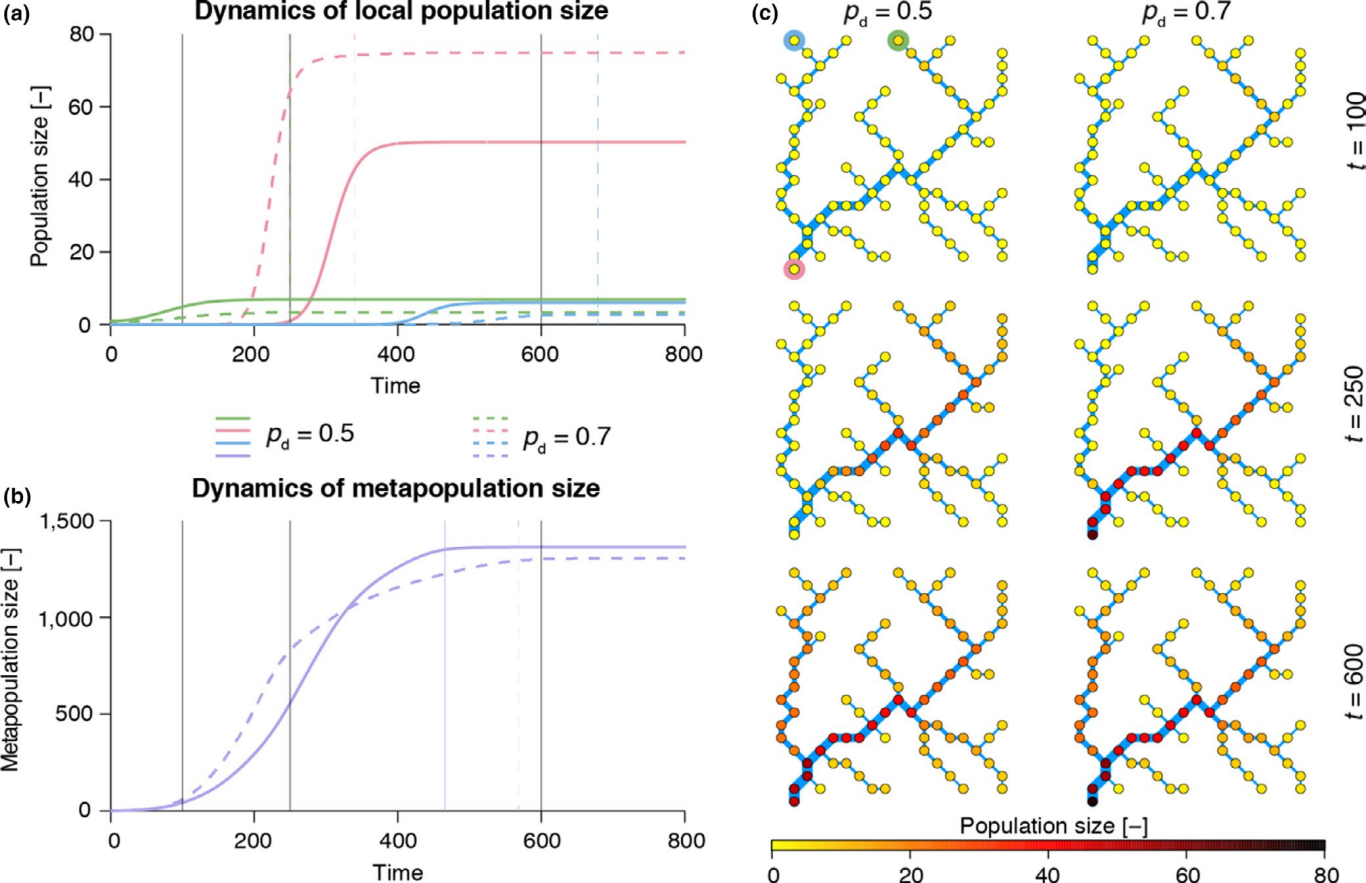
Results from the application of a metapopulaton model on an OCN. (a) Dynamics of local population size in three nodes: green represents the headwater that is invaded at the beginning of the simulation; red, the outlet; blue, the headwater that is farthest from the green node (see circles in the top‐left corner of panel c). Colored vertical lines represent the time steps when the respective local population has reached the equilibrium (arbitrarily imposed as 99% of the population size at t=800). Black solid lines identify the time steps used in panel (c). (b) Dynamics of the overall metapopulation size. Line styles as in panel (a). (c) Snapshots (obtained via *draw_thematic_OCN*) of spatial spread of the metapopulation at 3 different time steps

We performed two model simulations to investigate the effect of parameters *p_d_*, *p_u_* on the time elapsed until the system reaches a steady state; in a first (default) run, no preferential direction of movement was assumed (*p_d_* = *p_u_* = 0.5); in the second run, a preference for downstream movement (*p_d_* = 0.7, *p_u_* = 0.3) was hypothesized. Results are shown in Figure 7. When *p_d_* = 0.5, the invading species rapidly reaches the equilibrium in the initially occupied node, while colonization of the downstream patches is delayed. When a preference for downstream movement is attributed (*p_d_* = 0.7), local population growth in the onset (green) node is slower, whereas invasion of the outlet node occurs faster, both in terms of initial growth and establishment of the equilibrium (see colored vertical lines in Figure 7a). Colonization of the headwater that is farthest from the onset node is also delayed with respect to the default case. As a result, when *p_d_* = 0.7, the metapopulation size initially grows faster than when *p_d_* = 0.5, due to fast invasion of the downstream nodes and growth of the local populations therein (see Figure 7b); in a second phase, the growth rate of the metapopulation is reduced, because invasion of the upstream nodes is hampered by the low *p_u_* value, and the establishment of the equilibrium is delayed. As for the spatial spread of the metapopulation, when a preference for downstream movement is adopted, local population sizes at equilibrium tend to increase in the downstream nodes and decrease in the upstream nodes with respect to the default case (Figure 7a), resulting in a slightly lower overall metapopulation size at equilibrium (Figure 7b).

## 5 CONCLUSIONS

The importance of adequately representing spatial processes in ecological and evolutionary studies cannot be overstated. In the realm of freshwater ecology in particular, it is essential to consider how geomorphology shapes the structure of dendritic river networks and the ensuing connectivity configuration, which in turn control the variability of physical habitats and environmental variables, the dispersal of species and pathogens, and the spatial patterns of biodiversity and ecosystem processes. To this end, we presented *OCNet*, an R‐package that enables the generation of optimal channel networks, spanning trees that reproduce all scaling features of real river networks throughout the globe. These can be used as realistic riverine landscape analogues for a number of ecological, epidemiological, ecohydrological and evolutionary studies. We reviewed the theoretical background of the OCN concept and the existing applications on problems of ecological relevance, provided an overview of the main functionalities of the package, and proposed an example of application in the context of an invasive riverine species. We believe that this tool will allow a leap forward in the way spatial processes in river networks are investigated.

## CONFLICT OF INTEREST

The authors declare no conflict of interest.

## AUTHOR CONTRIBUTION

**Luca Carraro:** Formal analysis (lead); Investigation (lead); Methodology (equal); Software (lead); Visualization (lead); Writing‐original draft (lead); Writing‐review & editing (equal). **Enrico Bertuzzo:** Conceptualization (equal); Investigation (equal); Methodology (supporting); Software (supporting); Supervision (supporting); Writing‐review & editing (equal). **Emanuel A. Fronhofer:** Methodology (supporting); Software (supporting); Writing‐review & editing (equal). **Reinhard Furrer:** Methodology (supporting); Software (supporting); Writing‐review & editing (equal). **Isabelle Gounand:** Methodology (supporting); Software (supporting); Writing‐review & editing (equal). **Andrea Rinaldo:** Conceptualization (equal); Formal analysis (supporting); Methodology (supporting); Supervision (supporting); Writing‐review & editing (equal). **Florian Altermatt:** Conceptualization (equal); Funding acquisition (lead); Project administration (lead); Resources (lead); Supervision (lead); Writing‐original draft (supporting); Writing‐review & editing (equal).

## Data Availability

The code of the *OCNet* package is accessible on both CRAN (https://CRAN.R‐project.org/package=OCNet) and Github (http://doi.org/10.5281/zenodo.3669873 ‐ development version). A code script generating the figures of the manuscript can be found in http://github.com/lucarraro/OCNet‐ECE‐CreateFigures.
